# Early Detection of Alzheimer’s Disease Using Magnetic Resonance Imaging: A Novel Approach Combining Convolutional Neural Networks and Ensemble Learning

**DOI:** 10.3389/fnins.2020.00259

**Published:** 2020-05-13

**Authors:** Dan Pan, An Zeng, Longfei Jia, Yin Huang, Tory Frizzell, Xiaowei Song

**Affiliations:** ^1^School of Computers, Guangdong University of Technology, Guangzhou, China; ^2^Guangdong Key Laboratory of Big Data Analysis and Processing, Guangzhou, China; ^3^SFU ImageTech Lab, Surrey Memorial Hospital, Fraser Health, Surrey, BC, Canada

**Keywords:** Alzheimer’s disease, mild cognitive impairment, convolutional neural networks, ensemble learning, magnetic resonance imaging, MRI biomarkers, MCI-to-AD conversion, Alzheimer’s Disease Neuroimaging Initiative

## Abstract

Early detection is critical for effective management of Alzheimer’s disease (AD) and screening for mild cognitive impairment (MCI) is common practice. Among several deep-learning techniques that have been applied to assessing structural brain changes on magnetic resonance imaging (MRI), convolutional neural network (CNN) has gained popularity due to its superb efficiency in automated feature learning with the use of a variety of multilayer perceptrons. Meanwhile, ensemble learning (EL) has shown to be beneficial in the robustness of learning-system performance via integrating multiple models. Here, we proposed a classifier ensemble developed by combining CNN and EL, i.e., the CNN-EL approach, to identify subjects with MCI or AD using MRI: i.e., classification between (1) AD and healthy cognition (HC), (2) MCIc (MCI patients who will convert to AD) and HC, and (3) MCIc and MCInc (MCI patients who will not convert to AD). For each binary classification task, a large number of CNN models were trained applying a set of sagittal, coronal, or transverse MRI slices; these CNN models were then integrated into a single ensemble. Performance of the ensemble was evaluated using stratified fivefold cross-validation method for 10 times. The number of the intersection points determined by the most discriminable slices separating two classes in a binary classification task among the sagittal, coronal, and transverse slice sets, transformed into the standard Montreal Neurological Institute (MNI) space, acted as an indicator to assess the ability of a brain region in which the points were located to classify AD. Thus, the brain regions with most intersection points were considered as those mostly contributing to the early diagnosis of AD. The result revealed an accuracy rate of 0.84 ± 0.05, 0.79 ± 0.04, and 0.62 ± 0.06, respectively, for classifying AD vs. HC, MCIc vs. HC, and MCIc vs. MCInc, comparable to previous reports and a 3D deep learning approach (3D-SENet) based on a more state-of-the-art and popular Squeeze-and-Excitation Networks model using channel attention mechanism. Notably, the intersection points accurately located the medial temporal lobe and several other structures of the limbic system, i.e., brain regions known to be struck early in AD. More interestingly, the classifiers disclosed multiple patterned MRI changes in the brain in AD and MCIc, involving these key regions. These results suggest that as a data-driven method, the combined CNN and EL approach can locate the most discriminable brain regions indicated by the trained ensemble model while the generalization ability of the ensemble model was maximized to successfully capture AD-related brain variations early in the disease process; it can also provide new insights into understanding the complex heterogeneity of whole-brain MRI changes in AD. Further research is needed to examine the clinical implication of the finding, capability of the advocated CNN-EL approach to help understand and evaluate an individual subject’s disease status, symptom burden and progress, and the generalizability of the advocated CNN-EL approach to locate the most discriminable brain regions in the detection of other brain disorders such as schizophrenia, autism, and severe depression, in a data-driven way.

## Introduction

Alzheimer’s disease (AD) is a chronic, progressive, and irreversible neurodegenerative disease clinically manifested by amnesia, cognitive dysfunction, and gradual loss of multiple other brain functions and daily living independency (Ulep et al., 2018). The number of patients with AD worldwide is expected to increase from the current 47 million to 152 million by 2050, causing serious economic, medical, and societal consequences (Christina, 2018). The pathogenesis of AD remains not fully elucidated and no available therapy can cure AD or completely stop disease progression. Amnestic mild cognitive impairment (MCI) is a transitional stage between cognitively normal aging and AD, and patients with MCI are more likely to develop AD than age-matched healthy cognition (HC) (Liu et al., 2014). Early detection of AD by screening MCI is crucial both for effective management and care strategies and for developing new drugs and measures to prevent further deterioration of the disease.

Brain magnetic resonance imaging (MRI) has enabled non-invasive *in vivo* investigations of AD-related changes in the brain. A large number of promising machine learning applications have used MRI for AD prediction (Mateos-Pérez et al., 2018), which include random forests (RF) (Tripoliti et al., 2011), support vector machine (SVM) (Leemput et al., 2002), and boosting algorithms (Hinrichs et al., 2009). Even so, existing machine learning approaches typically involve manual selection of pre-defined brain regions of interest (ROIs) based on known MRI features of AD. Given the limited understanding of definitive MRI biomarkers for AD, it is likely that pre-selected ROIs cannot include all the information potentially useful to uncover the complexity of AD. Manual selection can also be prone to subjective errors and be time-consuming and labor-intensive (Li et al., 2018).

Deep learning represents a more advanced approach; methods such as stacked auto-encoder (SAE) (Vincent et al., 2010), deep belief networks (DBNs) (Hinton, 2009), and convolutional neural networks (CNNs) (LeCun, 2015) can automatically build a more abstract high-level representation of the learning system by integrating low-level features embedded in the data (Sun et al., 2012). The CNN model has been widely used for classification (Krizhevsky et al., 2012), segmentation (Long et al., 2015), and object detection (Girshick et al., 2014), due to several advantages: CNNs can directly accept images data as input, utilize spatial information embedded in adjacent pixels, and effectively reduce the number of model parameters by using local receptive fields, weights sharing, and subsampling. When a CNN model is trained with MRI slices, image features can be automatically retrieved, eliminating the need of manual selection of features for the learning process (Lin et al., 2018). Meanwhile, ensemble learning (EL) has shown beneficial in the performance and robustness via integrating multiple learning systems (Opitz and Maclin, 1999), which has also been applied to MRI (Ortiz et al., 2016).

So far, some researchers have combined deep learning and EL on MRI data for AD. A method for AD and early AD diagnosis by fusing functional and structural imaging data based on the use of the Deep Learning paradigm, and more specifically, deep belief networks (DBN) has been advocated (Ortiz et al., 2016). Gray matter (GM) images from each brain area have been split into 3D patches according to the regions defined by the Automated Anatomical Labeling (AAL) atlas, and these patches were used to train a set of DBNs. The DBNs were then ensembled where the final prediction was determined by a voting scheme. Two deep learning based structures and four different voting schemes were implemented and compared, giving as a result a potent classification architecture where discriminative features were computed in an unsupervised fashion (Ortiz et al., 2016). Islam and Zhang (2018) proposed an ensemble of three deep CNNs with slightly different configurations for Alzheimer’s disease diagnosis using brain MRI data analysis. In addition, sparse regression models were combined with deep neural networks for AD diagnosis (Suk et al., 2017). Here, sparse regression models with different regularization control values outputted their own prediction values. To obtain the final prediction values, CNNs discovered the optimal weights to ensemble multiple sparse regression models in a hierarchical and non-linear way (Suk et al., 2017). In 2019, 20 white matter and GM slices with significant brain structures from MR images were selected to train an ensemble of ConvNet networks (Ji et al., 2019). In Li et al. (2018), a whole MR brain image was partitioned into different local regions and a number of 3D patches were extracted from each region. Subsequently, the authors grouped the patches from each region into different clusters with the K-Means clustering method. Next, a DenseNet was constructed to learn the patch features for each cluster and the features acquired from the discriminative clusters of each region were ensembled for classification. At the end, the authors combined the classification results from different local regions to improve final image classification.

In the present study, we proposed a novel CNN–EL approach based on an established eight-layer CNN network structure (Wang et al., 2018), to automatically retrieve features from brain MRI data that can be used to differentiate subjects with clinical diagnosed AD and MCI from HC, and those with MCIc and MCInc. We are also interested in identifying patterns of MRI brain changes that characterize AD and MCIc. To achieve the study objectives, we first derived a CNN model using each set of the sagittal, coronal, or transverse MRI slices; then, we developed a classifier ensemble based on three-axis slices using EL. A number of sophisticated techniques were employed in our approach, which included six ways of data augmentation (DA) to facilitate an equal and relatively large number of instances of each class in the training dataset, top-performance enforcing to achieve a high classification accuracy and robustness of the model training, and parallel processing to improve the time efficiency of the system function.

In the CNN-EL, a data-driven, homogeneous ensemble learning approach was employed. A base classifier based on 2D CNN model was trained using each set of the sagittal, coronal, or transverse MRI slices; that is, a trained base classifier corresponds to a slice dataset, which is composed of slices in a specific position in brain from the subjects in the training dataset. The preparations of training datasets didn’t depend on prior experience or domain knowledge. In order to reduce the loss of information as much as possible during the process of slicing the 3D volume into 2D slices, we have utilized as many and meaningful 2D-sagittal, -coronal, or -transverse slices from all over the brain as we can at the same time to train the base classifiers. Among them, the trained base classifiers with the best generalization performance on the validation datasets were selected and combined to generate a refined final classifier ensemble based on three-axis slices. In this data-driven way, the slices corresponding to the selected trained base classifiers were considered as those with the strongest capabilities to classify AD. The number of the intersection points determined by the most discriminable slices separating two classes in a binary classification task among the sagittal, coronal, and transverse slice-sets, transformed into the standard Montreal Neurological Institute (MNI) space, acted as an indicator to assess the ability of a brain region in which the points were located to classify AD. Thus, we located the most discriminable brain regions indicated by the trained CNN-EL model while its generalization abilities were maximized and superior to those of the compared methods. That is, we can understand the predictions made by the trained CNN-EL model to some extent. However, the compared methods, i.e., PCA + SVM (Christian et al., 2015) and a 3D deep learning approach (3D-SENet) based on a more state-of-the-art and popular Squeeze-and-Excitation Networks model using channel attention mechanism, which was derived from the paper (Hu et al., 2018), were unable to do the same thing as the above-mentioned and failed to provide meaningful explanations for predictions since the models achieved with those compared methods were still like a “black-box”. To our knowledge, this is the first attempt to do the above way with both CNN and EL, and at the same time, the promising experimental results have been achieved.

In detail, the CNN-EL was different from the above-mentioned methods which combined the deep learning with the ensemble learning to analyze MRI data for detecting AD in the base classifiers (Ortiz et al., 2016; Suk et al., 2017; Islam and Zhang, 2018; Li et al., 2018), the ensemble methods (Ortiz et al., 2016; Suk et al., 2017; Islam and Zhang, 2018), the model interpretability (Ortiz et al., 2016; Suk et al., 2017; Islam and Zhang, 2018), or the preparation of training datasets (Ortiz et al., 2016; Li et al., 2018; Ji et al., 2019).

Furthermore, in the paper (Wen et al., 2019), the authors firstly systematically and critically reviewed the state-of-the-art on classification of Alzheimer’s disease based on convolutional neural networks and T1-weighted MRI. Next, they proposed an open-source framework for reproducible evaluation of classification approaches. In this study, the fivefold cross validation procedure was strictly followed and repeated ten times for each binary experiment, i.e., AD vs. HC, MCIc vs. HC, and MCIc vs. MCInc. The potential data leakage among binary classification tasks was avoided and therefore the experimental results were unbiased and reproducible.

## Materials and Methods

### Participants and Datasets

Data used in the study were obtained from the Alzheimer’s Disease Neuroimaging Initiative (ADNI) database.^1^ The ADNI was launched in 2003 as a public–private partnership, led by Principal Investigator, Michael W. Weiner, MD. The primary goal of ADNI has been to test whether serial MRI, positron emission tomography (PET), other biological markers, and clinical and neuropsychological assessment can be combined to measure the progression of MCI and early AD.

To facilitate comparison of our results with those reported previously, we used the same MRI dataset from the ADNI database as utilized by Christian et al. (2015) in building the eight-layer CNN networks (Wang et al., 2018) to train the base classifiers, as well as to test the performance of the final classifier ensemble based on three-axis slices (*n* = 509 subjects: *AD* = 137, 18 months MCIc = 76 and MCInc = 134, and *HC* = 162; Table 1A). We enrolled 162 cognitively normal elderly controls (HC), 137 patients with diagnosis of AD, 76 patients with diagnosis of MCI who converted to AD within 18 months (MCIc), and 134 patients with diagnosis of MCI who did not convert to AD within 18 months (MCInc). MCI patients who had been followed less than 18 months were not considered (Christian et al., 2015). A total of 509 subjects from 41 different radiology centers were considered. Inclusion criteria for HC were as follows: Mini Mental State Examination (MMSE) scores between 24 and 30; Clinical Dementia Rating (CDR) (Morris, 1993) of zero; and absence of depression, MCI, and dementia. Inclusion criteria for MCI were as follows: MMSE scores between 24 and 30; CDR of 0.5; objective memory loss, measured by education adjusted scores on Wechsler Memory Scale Logical Memory II (Wechsler, 1987); absence of significant levels of impairment in other cognitive domains; and absence of dementia. Inclusion criteria for AD were as follows: MMSE scores between 20 and 26; CDR of 0.5 or 1.0; and NINCDS/ADRDA criteria for probable AD (McKhann et al., 1984; Dubois et al., 2007).

**TABLE 1:** Characteristics of participants in **(A)** the training and testing dataset (upper panel) and **(B)** the validation dataset (lower panel).

Variable	AD	MCIc	MCInc	HC
**(A)**				
*N*	137	76	134	162
Gender (male:female)	67:70	43:33	84:50	86:76
Age (year; mean, std)	76.0,7.3	74.8, 7.3	74.5, 7.2	76.3, 5.4
Weight (kg; mean, std)	70.9, 14.0	72.7, 14.3	76.2, 12.9	73.8, 13.6
MMSE (mean, std)	23.2, 2.0	26.47, 1.84	27.19, 1.71	29.18, 0.96
CDR (mean, std)	0.75, 0.25	0.50, 0.00	0.50, 0.00	0.00, 0.00
GDS (mean, std)	1.59, 1.32	1.38, 1.14	1.52, 1.37	0.80, 1.08
**(B)**				
*N*	100	39	39	100
Gender (male:female)	60:40	23:16	29:10	45:55
Age (years; mean, std)	74.24, 7.82	74.15, 7.10	76.02, 7.00	73.36, 5.70
Weight (kg; mean, std)	76.04, 15.83	73.59, 14.14	78.35, 12.99	76.16, 15.66
MMSE (mean, std)	23.84, 2.08	27.05, 1.59	27.56, 1.83	28.92, 1.25
CDR (mean, std)	0.82, 0.24	0.50, 0.00	0.50, 0.00	0.00, 0.00
GDS (mean, std)	1.81, 1.56	1.92, 1.35	1.79, 1.45	0.83, 1.34

*AD, Alzheimer’s disease patients; MCIc, mild cognitive impairment patients who will convert to AD; MCInc, mild cognitive impairment patients who will not convert to AD; HC, healthy controls; MMSE, Mini Mental State Examination; CDR, Clinical Dementia Rating; GDS, Global Deterioration Scale.*

To facilitate the development of the EL process, an additional validation dataset of 278 subjects (*AD* = 100, 36 months MCIc = 39 and MCInc = 39, and *HC* = 100; Table 1B) was also retrieved from the ADNI database and used to identify the base classifiers showing the best generalization performance. The validation data of 278 subjects had no overlapping with the aforementioned data of 509 subjects, i.e., the validation data were used for neither training the base classifiers nor testing the acquired final classifier ensemble based on three-axis slices (Table 1B). Here, among 164 patients with diagnosis of pMCI (progressive MCI) used by Moradi et al. (2015), i.e., if diagnosis was MCI at baseline but conversion to AD was reported after baseline within 1, 2, or 3 years, and without reversion to MCI or HC at any available follow-up (0–96 months), 39 patients who were not in the 509 subjects were selected as MCIc subjects in the validation dataset. Meanwhile, among 100 patients with diagnosis of sMCI (stable MCI) used by Moradi et al. (2015), i.e., if diagnosis was MCI at all available time points (0–96 months) but at least for 36 months, 39 patients who were not in the aforementioned 509 subjects were chosen as MCInc subjects in the validation dataset. In order to keep the validation dataset relatively balanced, we enrolled 100 cognitively normal elderly controls (HC) and 100 patients with diagnosis of AD who were not in the aforementioned 509 subjects as well.

### MRI Preprocessing

**FIGURE 1:**
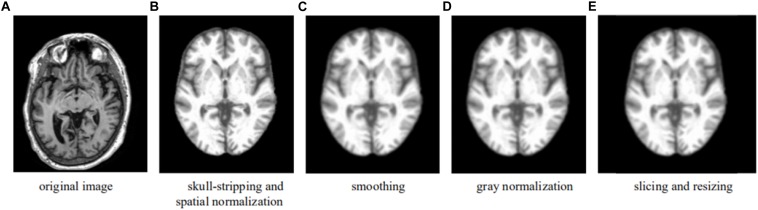
Preprocessing pipeline—an example showing the formation of a transverse slice used in the learning. **(A)** Original image. **(B)** Skull-stripping and spatial normalization. **(C)** Smoothing. **(D)** Gray normalization. **(E)** Slicing and resizing.

Upon downloading, the T1-weighted MRI data in.nii format were processed using the CAT12 toolkit^2^ with default value setting. The preprocessing pipeline included skull extraction, registration to the MNI space, and image smoothing, so that after processing, all the images had a dimension of 121 × 145 × 121 (*X* × *Y* × *Z*) with a spatial resolution of 1.5 × 1.5 × 1.5 mm^3^ per voxel. Voxel-based MRI signal intensity normalization was then performed for each image; i.e., the value of each voxel was normalized as the original value divided by the original maximal value of the image, yielding a value between 0 and 1. The complete preprocessing pipeline is summarized in Figure 1.

**FIGURE 2:**
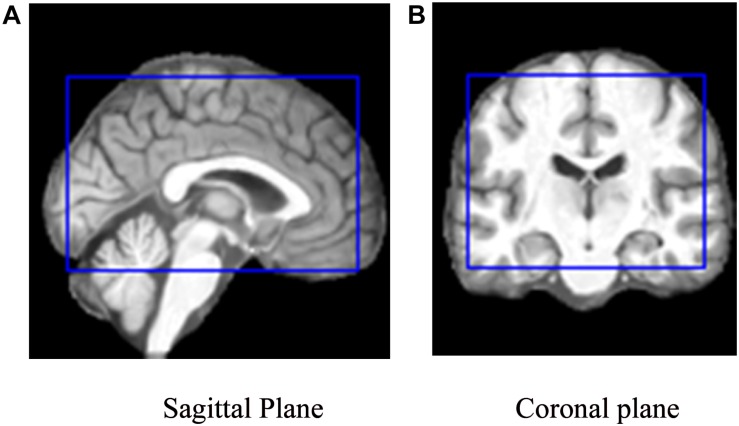
The cropping range (inside the blue rectangle) of the slices used to train the model on **(A)** a sagittal plane and **(B)** a coronal plane, respectively. **(A)** Sagittal Plane. **(B)** Coronal plane.

To facilitate the CNN training, verification, and testing, a 3D image set of each subject was re-sliced into three 2D image sets, each of the sagittal, coronal, or transverse orientation (with *X*, *Y*, and *Z* axes perpendicular to the sagittal, coronal, and transverse planes, respectively). A preprocessed 3D MRI image (of 121 × 145 × 121) was thus re-sliced into 121 sagittal, 145 coronal, and 121 transverse slices; the values on the *X*, *Y*, and *Z* axis were {−90, −88, −87, … 90}, {−126, −125, −123, … 90}, and {−72, −71, −69, … 108}, respectively. For example, *X*(*i*), *i*∈{−90, −88, −87, … 90} is the sagittal slice through the point [*i*, 0, 0]. Here, the numbers within the brackets were the MNI coordinates. To reduce the number of base classifiers without compromising the effectiveness of the classification, every other slice was used (given the relatively small difference between two adjacent slices) and slices near either end of an axis were discarded (given the relatively less amount of information useful for classification), which lay outside the blue rectangle shown in Figure 2. The CNN model training, testing, and verification involved use of only 40 sagittal slices {*X*(−61), *X*(−58), *X*(56)}, 50 coronal slices {*Y*(−91), *Y*(−88), *Y*(56)}, and 33 transverse slices {*Z*(−28), *Z*(−25), *Z*(68)}, i.e., in total, 123 slices of a subject’s 3D brain image.

Given the dimension of the 3D MRI (121 × 145 × 121), the sizes of the sagittal, coronal, and transverse slices obtained through re-slicing were 145 × 121, 121 × 121, and 121 × 145, respectively. Each of the 2D slices was reformatted to 145 × 145 using edge padding and zero filling, so that the 2D slice is squared, while the center and the spatial resolution of the resized image remained unchanged.

### Convolutional Neural Network

As an automated image recognition method, the CNN has attracted widespread research attention with tremendous success in recent years. Hubel and Wiesel first described receptive fields, binocular interactions, and the functional architecture of cat primary visual cortex about 55 years ago (Hubel and Wiesel, 1962, 1965). Kunihiko Fukushima proposed a neural network model nicknamed “Neocognitron” (Fukushima, 1980) that is structurally similar to the hierarchy model of the visual nervous system proposed by Hubel and Wiesel. This unique network structure can effectively reduce the complexity of feedback neural networks, which characterizes the CNN model. With the CNN, each input image is passed through a series of convolution layers: filtering layers (kernels), pooling layers, and fully connected layers (FCs). A softmax function is then applied to classify an image with probabilistic values between 0 and 1, making the CNN suitable for learning representations of image features (Schmidhuber, 2015).

**FIGURE 3:**
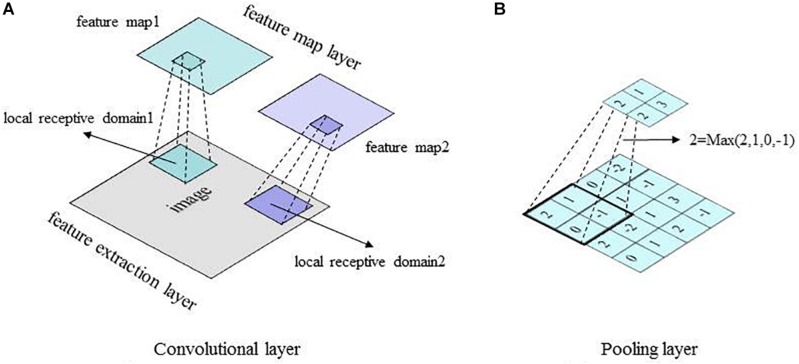
Basic structures of the CNN convolutional layer and pooling layer. **(A)** Convolutional layer. **(B)** Pooling layer.

A convolution layer in the CNN model is typically composed of two segments: feature extraction and feature mapping (Krizhevsky et al., 2012). In the feature-extraction segment, each neuron is connected to the local receptive field of the upper layer to extract local features. Once the local feature is extracted, its spatial relationship with other features is also determined. In the feature-mapping segment, convolution is performed on the input data using a learnable filter or kernel to produce a feature map. Feature mapping computes the outputs of neurons connected to receptive fields in the input, with each neuron computing a dot product between its weight (i.e., filter) and a local receptive field (equivalent to filter size) to which it is connected (the input volume). Multiple feature maps can be calculated with a set of learnable filters. In this way, the number of parameters to be tuned in the CNN is effectively reduced. A convolutional layer is followed by a pooling layer, e.g., max-pooling layer (Weng et al., 1992), which performs a down-sampling operation along the spatial dimensions (e.g., *X*, *Y* for a transverse slice). This unique dual-feature extraction method can effectively reduce the feature resolution (Krizhevsky et al., 2012). The basic structures of the convolutional layer and the pooling layer of the CNN model are shown in Figure 3.

In this study, the CNN was utilized mainly to recognize 2D images with displacement, scaling, and other non-deformed distortions. Data were reconstructed, so that an image was inputted into the CNN model as a vector for easy feature extraction and classification. The effectiveness of the CNN was improved as the pooling layer learned the features from training data without manual extraction. Applying the learnable kernels and convolution operation, the CNN was trained in parallel, while the local weight-sharing effectively reduced its complexity.

### Ensemble Learning

EL algorithms including Bagging (Breiman, 1996), Boosting (Freund and Schapire, 1997), and Random Forest (Breiman, 2001) have been typically used to construct a set of base classifiers in solving a given problem. Using a training dataset, EL discriminates features to produce a weighted vote for classes, which is then applied in classifying more cases in new datasets. Based on the methods with which a base learner is generated, each of the EL algorithms can be divided into two general approaches: the heterogeneous approach, which applies different learning algorithms in the same training data, and the homogeneous approach, which applies the same learning algorithm in different training data (Zhang and Zhang, 2011). Both approaches have been shown to significantly improve the generalizability and robustness of a learning system.

In the present study, the homogeneous EL approach was adopted from the stratified Bagging method. The same CNN algorithm was employed to train different base classifiers using different 2D MRI slices. The outputs from the multiple trained base classifiers with the best generalization performance on the validation dataset were then combined to generate a refined final classifier ensemble based on three-axis slices that was used to predict classification results for new cases, i.e., 3D MRI data.

### Classification Experiment

A total of 787 subjects’ 3D MR images from the ADNI database were partitioned into three datasets: training and testing datasets to build the base classifiers and examine the performance of the final classifier ensemble based on three-axis slices (*n* = 509; Table 1A) and a verification dataset to evaluate and select the best base classifiers (*n* = 278; Table 1B). For training and testing, a stratified fivefold cross-validation method was employed, such that each binary classification task (e.g., MCIc vs. MCInc) was conducted five times. No images in the training/testing datasets were used to select the best base classifiers, and thus potential data leakage among binary classification tasks was avoided.

In each binary classification task, a total of 123 2D sagittal, coronal, and transverse slices extracted from each 3D MRI were employed to generate 123 trained base classifiers. Using classification of AD (*n* = 137) vs. HC (*n* = 162) as an example, 299 labeled 3D MRI (Table 1A), were partitioned into 80% training and 20% testing cases with stratified random sampling. The 299 2D slices of *X*(*i*) [or *Y*(*j*), or *Z*(*k*)] were compiled as a 2D dataset, where *i*∈{−61, −58,…56}, *j*∈{−91, −88,…56}, and *k*∈{−28, −25,…68}; 239 (or 80%) of stratified randomly selected cases were employed to train the *X*(*i*) [or *Y*(*j*), or *Z*(*k*)] base classifier, while the remaining slices of 60 (or 20%) cases were used to test the trained classifier ensemble based on three-axis slices. In this way, all 123 trained base classifiers to classify AD vs.HC were acquired.

Then, the 123 labeled 2D MR images from each of AD (*n* = 100) and HC (*n* = 100) cases were altogether used as the validation dataset (Table 1B): they were employed to select the five base classifiers (i.e., in total 15) with the best generalization performance, as determined by classification accuracy, among the sagittal, coronal, and transverse slice-based base classifiers, respectively. The number of five was determined by the experiments. Finally, after building three classifier ensembles based on single-axis slices (i.e., sagittal, coronal, and transverse), a classifier ensemble based on three-axis slices, which was composed of all the three classifier ensembles based on single-axis slices, was finally built using these 15 base classifiers, following a simple majority voting scheme (Arora et al., 2012). The 2D slices that were extracted from the 3D MR images of the remaining 60 (or 20%) cases in the training and testing dataset and were corresponding to the 15 base classifiers were used to test the performance of the built classifier ensemble based on three-axis slices.

### Data Augmentation

To overcome the possible over-fitting problem in training robust CNN models and to incorporate possible image discrepancy, augmented images were generated from the original slices by six operations: rotation, translation, gamma correction, random noise addition, scaling, and random affine transformation. The augmented data were added to the original training dataset to allow a sufficiently large sample size (Table 2). Data augmentation was also used to mitigate the originally imbalanced dataset (e.g., there were more subjects with MCInc than those with MCIc), for which the preset number of augmented slices to be generated varied from class to class. For example, to classify MCIc vs. MCInc, there were 76 MCIc and 134 MCInc cases. Using six data augmentation operations, 10 new slices were generated from an MCInc case and 18 from an MCIc case with each operation. In this way, slice ratios of MCInc:MCIc became ∼1:1 after data augmentation from the original ∼1.8:1.

**TABLE 2:** Numbers of augmented images in the MCIc and MCInc datasets.

Augmentation methods	MCIc	MCInc	Total
Original slices	76	134	210
Rotation	1368	1340	2708
Translation	1368	1340	2708
Gamma correction	1368	1340	2708
Random noise	1368	1340	2708
Scaling	1368	1340	2708
Random affine transformation	1368	1340	2708
Total number of images in the augmented dataset	8284	8174	16,458

## Results

### Base Classifiers

**FIGURE 4:**
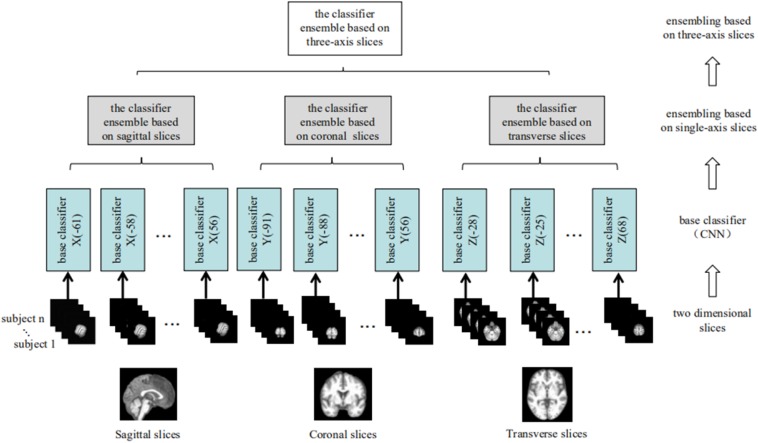
The architecture of the classifier ensemble based on the three sets of 2D slices (from left to right: sagittal, coronal, and transverse).

**FIGURE 5:**
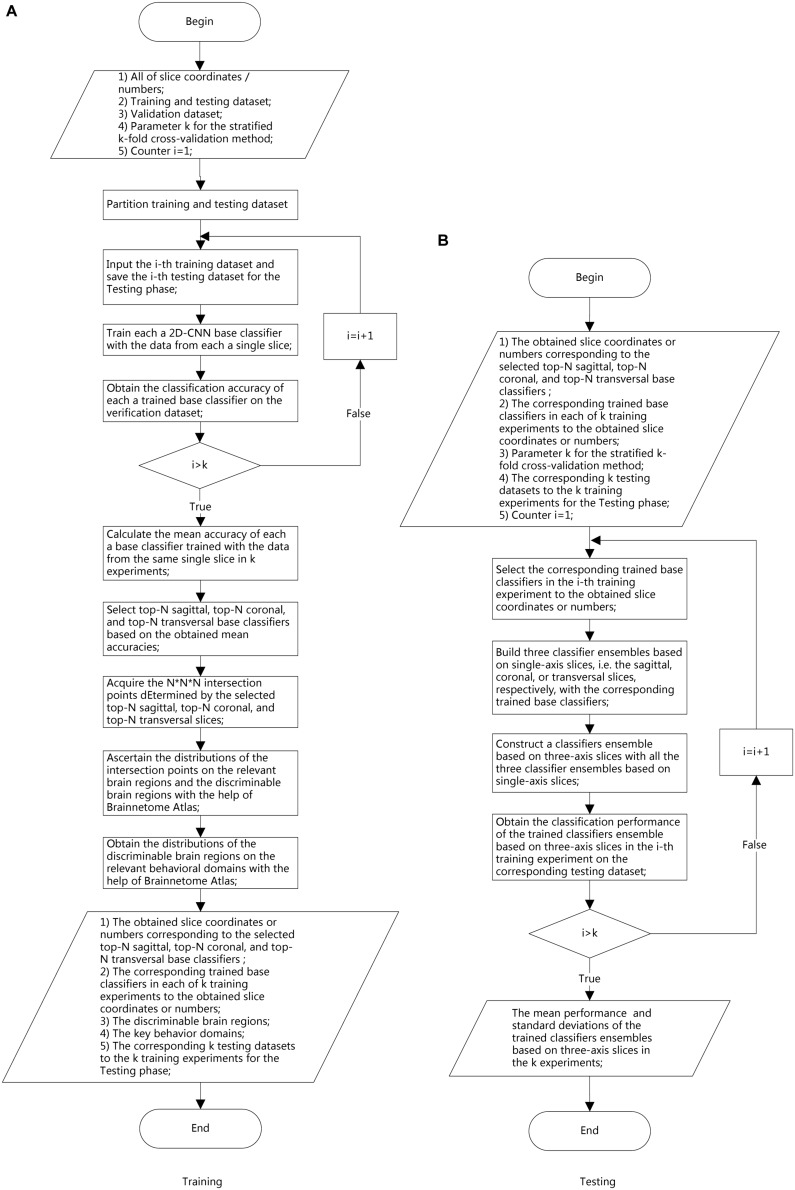
Experimental flow chart. **(A)** Training phase. **(B)** Testing phase.

To address the objective of the study, i.e., binary classification of AD or MCIc vs. HC, and MCIc vs. MCInc, three corresponding classifier ensembles based on the three slice orientation groups (sagittal, coronal, and transverse), i.e., classifier ensembles based on three-axis slices, were trained. The overall architecture of the proposed classifier ensemble based on three-axis slices is shown in Figure 4 and the flow chart of the experiment is shown in Figure 5.

**FIGURE 6:**
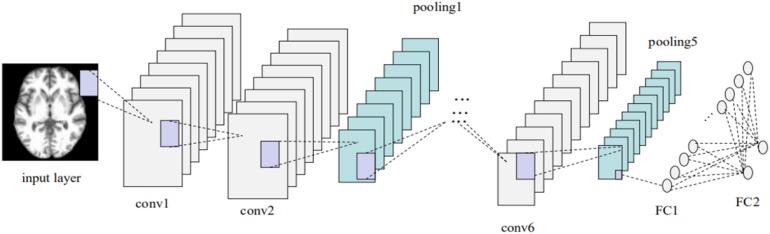
Base-classifier architecture used in the CNN-EL approach proposed here.

Each base classifier consisted of six convolution layers (conv) and two fully connected layers (FCs). The last FC layer had only two nodes, and the softmax function was used to implement the binary classification. The network architecture and corresponding hyper-parameters are shown in Figure 6 and Table 3, respectively. Each base classifier was trained for 30 epochs, as 30 epochs proved sufficient for a base classifier to converge. That is, after 30 epochs, a trained base classifier could achieve 100% classification accuracy on the original slices (rather than the augmented slices) in the training dataset. Activation functions in all convolutional layers were of the leaky rectifier linear activation (LReLU) type (Shan et al., 2016), while the Adam optimization algorithm (Kingma and Ba, 2014) was used to update network weights. The learning rate and the batch size were set to 0.0001 and 200, respectively.

**TABLE 3:** Detailed hyper-parameters of base classifiers of the CNN-EL approach advocated here.

Layer #	Layer name	Kernel size	Strides	Input channels	Output channels
1	conv1	3*3	3	1	32
2	conv2	3*3	3	32	64
	pooling1	3*3	1	N/A	N/A
3	conv3	3*3	3	64	128
	pooling2	3*3	1	N/A	N/A
4	conv4	1*1	1	128	256
	pooling3	3*3	1	N/A	N/A
5	conv5	1*1	1	256	512
	pooling4	3*3	1	N/A	N/A
6	conv6	1*1	1	512	1024
	pooling5	3*3	3	N/A	N/A
7	FC1	N/A	N/A	4096	100
8	FC2	N/A	N/A	100	2

### Ensemble Learning

The proposed model employed a two-stage EL scheme. Phase 1 involved building three classifier ensembles based on single-axis slices (i.e., sagittal, coronal, and transverse) and Phase 2 involved constructing a classifier ensemble based on three-axis slices, which was composed of all the three classifier ensembles based on single-axis slices acquired in Phase 1. In total, 40 sagittal, 50 coronal, and 33 transverse base classifiers were acquired. Then, the five base classifiers with the best generalization performance for each slice orientation were selected using the verification dataset, yielding three classifier ensembles based on single-axis slices, each with the 5 best base classifiers. The output of a classifier ensemble based on single-axis slices was generated by combining the outputs of the five best base classifiers. Finally, a simple majority voting scheme was used to combine the predictions of these three classifier ensemble based on single-axis slices to yield the output of the classifier ensemble based on three-axis slices. Experimental results demonstrated that this EL method greatly improved the generalizability and robustness of early stage AD detection.

### Classification Performance

Using the stratified fivefold cross-validation procedure and repeating it 10 times, the average classification accuracies were 84% for AD vs. HC, 79% for MCIc vs. HC, and 62% for MCIc vs. MCInc. The average classification accuracies for AD vs. HC and MCIc vs. HC were statistically significantly higher than those achieved using principal component analysis (PCA) plus the SVM method described in a previous study (Christian et al., 2015), while the average classification accuracy for MCIc vs. MCInc was not statistically significantly lower (Christian et al., 2015). As for the reason why the classification accuracy for MCIc vs. MCInc task was relatively low, we suppose the performance of the proposed CNN-EL method, as a deep learning approach, which usually demands more training data, was a little bit more negatively affected by the insufficient training samples in the MCIc vs. MCInc classification task. Plus, one additional possible reason might be the cutoff threshold of follow-up duration to define MCIc and MCInc, and the cohorts of MCIc and MCInc subjects might be highly heterogeneous regardless of the threshold used (Li et al., 2019).

More importantly, the standard deviations of the classification accuracies were only 0.05 for AD vs. HC, 0.04 for MCIc vs. HC, and 0.06 for MCIc vs. MCInc, all of which were about one-third of those reported previously (Christian et al., 2015).

In this study, all of the experiments were run on one node in a GPU cluster with five nodes, each of which had two NVIDIA Tesla P100-PCIe-16GB 250W cards. For a 1 × 5-fold cross-validation process, the computing time of the CNN-EL proposed here in AD vs. HC, MCIc vs. HC, and MCIc vs. MCInc task was about 21, 19, and 15 h, respectively.

**FIGURE 7:**
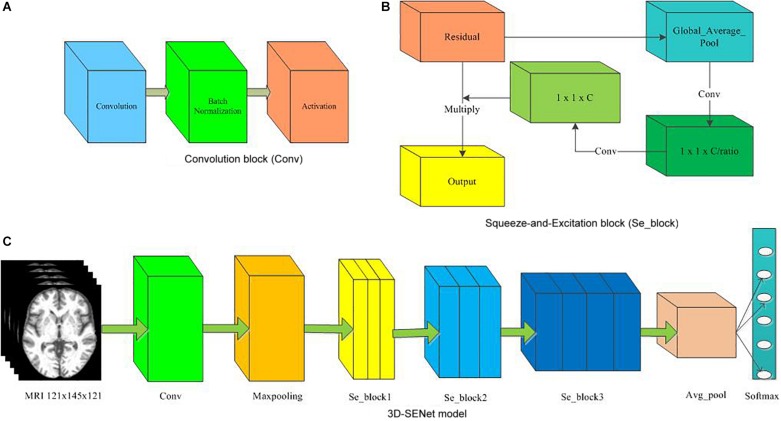
The architecture of the 3D-SENet model. **(A)** Convolution block (Conv), **(B)** Squeeze-and-Excitation block (Se_block), **(C)** 3D-SENet model.

At the same time, the proposed approach here was compared with the 3D-SENet. As the central building block of CNNs, the convolution operator could enable networks to acquire informative features by fusing both spatial and channel-wise information within local receptive fields at each layer. To achieve better generalization performance, the SENet automatically learned the weight of each feature channel to enhance the useful features and suppress the useless features for the task to be tackled, by introducing “Squeeze-and-Excitation” block as a self-attention function on channels (Hu et al., 2018). Here, the architecture of the compared 3D-SENet model and corresponding detailed hyper-parameters are shown in Figure 7 and Table 4, respectively. With 10 × 5-fold cross-validation processes, the accuracy rates of 0.80 ± 0.05, 0.75 ± 0.07, and 0.57 ± 0.11 were obtained, respectively, for classifying AD vs. HC, MCIc vs. HC, and MCIc vs. MCInc. For a 1 × 5-fold cross-validation process, the computing time of the 3D-SENet in AD vs. HC, MCIc vs. HC, and MCIc vs. MCInc task was about 11.5, 10.9, and 10.6 h, respectively.

**TABLE 4:** Detailed hyper-parameters of 3D-SENet model.

Layer name	Sub-layer name	Kernel size	Strides	Filters	Output size
MRI_images	N/A	N/A	N/A	N/A	121 × 145 × 121
Conv	N/A	3 × 3 × 3	1	64	121 × 145 × 121
Maxpooling	N/A	3 × 3 × 3	2	N/A	60 × 72 × 60
Se_block1	Block1	3 × 3 × 3	2	256	30 × 36 × 30
Se_block1	Block2	3 × 3 × 3	1	256	30 × 36 × 30
Se_block1	Block3	3 × 3 × 3	1	256	30 × 36 × 30
Se_block2	Block1	3 × 3 × 3	2	512	15 × 18 × 15
Se_block2	Block2	3 × 3 × 3	1	512	15 × 18 × 15
Se_block2	Block3	3 × 3 × 3	1	512	15 × 18 × 15
Se_block3	Block1	3 × 3 × 3	2	1024	8 × 9 × 8
Se_block3	Block2	3 × 3 × 3	1	1024	8 × 9 × 8
Se_block3	Block3	3 × 3 × 3	1	1024	8 × 9 × 8
Se_block3	Block4	3 × 3 × 3	1	1024	8 × 9 × 8
Avg_pool	N/A	8 × 9 × 8	1	1024	1 × 1 × 1024
Softmax	N/A	N/A	N/A	N/A	2

In order to evaluate the classification performance more comprehensively, the Area Under the Curve (AUC) and Matthews Correlation Coefficient (MCC) (Matthews, 1975) have been used as the performance metrics in this study as well. To verify whether or not our performance is different from those of two methods, i.e., Christian et al. (2015) and the 3D-SENet model, we have further run six hypothesis tests (*p*-value approach) for three binary experiments, i.e., AD vs. HC, MCIc vs. HC, and MCIc vs. MCInc. After the homogeneity of variance test was performed, the Student’s *t-*test with the Cox-Cochran correction for unequal variances was applied if the homogeneity of variance test failed. Experimental results and corresponding statistical performance comparisons with *p-*values are summarized in Tables 5, 6, respectively. For all three binary classification tasks, the average classification accuracies of the CNN-EL were statistically significantly higher than those achieved using the 3D-SENet, while the standard deviations of the CNN-EL were lower than or equal to those of the 3D-SENet.

**TABLE 5:** Comparison of experimental results with PCA + SVM (Christian et al., 2015) and 3D-SENet.

Experiment model	AD vs. HC			MCIc vs. HC			MCIc vs. MCInc		
			
	ACC	AUC	MCC	ACC	AUC	MCC	ACC	AUC	MCC
PCA + SVM	0.76 ± 0.11	–	–	0.72 ± 0.12	–	–	0.66 ± 0.16	–	–
3D-SENet	0.80 ± 0.05	0.88 ± 0.04	0.62 ± 0.09	0.75 ± 0.07	0.79 ± 0.07	0.42 ± 0.16	0.57 ± 0.11	0.57 ± 0.08	0.11 ± 0.15
CNN + EL proposed here	0.84 ± 0.05	0.92 ± 0.03	0.68 ± 0.10	0.79 ± 0.04	0.83 ± 0.06	0.49 ± 0.12	0.62 ± 0.06	0.59 ± 0.07	0.10 ± 0.15

**TABLE 6:** Statistical comparisons with *p-*values about accuracy mean of the three methods for **(A)** AD vs. HC task (upper panel), **(B)** MCIc vs. HC task (middle panel), and **(C)** MCIc vs. MCInc task (lower panel).

Model	PCA + SVM	3D-SENet	CNN + EL proposed here
**(A)**			
PCA + SVM	N/A	*p* > 0.05	*p* < 0.05
3D-SENet	*p* > 0.05	N/A	*p* < 0.05
CNN + EL proposed here	*p* < 0.05	*p* < 0.05	N/A
**(B)**			
PCA + SVM	N/A	*p* > 0.05	*p* < 0.05
3D-SENet	*p* > 0.05	N/A	*p* < 0.05
CNN + EL proposed here	*p* < 0.05	*p* < 0.05	N/A
**(C)**			
PCA + SVM	N/A	*p* < 0.05	*p* > 0.05
3D-SENet	*p* < 0.05	N/A	*p* < 0.05
CNN + EL proposed here	*p* > 0.05	*p* < 0.05	N/A

It can be seen that the proposed early detection model for Alzheimer’s disease based on CNN and EL was more accurate and robust than the PCA plus SVM method (Christian et al., 2015) and the 3D-SENet model.

### Discriminable Brain Regions

In the first phase of EL, the validation set was employed to examine each base classifier and subsequently to acquire three classifier ensembles based on each of the three single-axis slice datasets, each comprising of the best five sagittal, coronal, and transverse base classifiers in generalization capabilities. As a base classifier corresponds to a slice dataset, all 15 best base classifiers correspond to 15 slices in the *X*–*Y*–*Z* coordinate system, which can define 5 × 5 × 5 points in the *X*–*Y*–*Z* coordinate system. As an example, the sagittal, coronal, and transverse slice numbers corresponding to the 15 best base classifiers for the first time to run the stratified fivefold cross-validation procedure are shown in Table 7.

**TABLE 7:** Sagittal, coronal, and transverse slice numbers corresponding to the 15 best base classifiers for the first time to run the stratified fivefold cross-validation procedure.

Experiment	Rank	Sagittal slice #	Coronal slice #	Transverse slice #
AD vs. HC	1	22	−5	−23
	2	20	−17	−25
	3	16	−13	−19
	4	−20	−23	−17
	5	28	−7	−11
MCIc vs. HC	1	16	−13	−17
	2	20	−7	−25
	3	14	−11	−7
	4	26	−31	−23
	5	−20	−5	−29
MCIc vs. MCInc	1	−46	−13	−23
	2	−16	7	−19
	3	−44	−1	55
	4	−56	−79	−25
	5	−50	−35	−29

Take the AD vs. HC classification task for the first time to run the stratified fivefold cross-validation procedure as an example. One hundred twenty-five points in the *X*–*Y*–*Z* coordinate system were determined by the top 5 sagittal, coronal, and transverse slices, respectively, e.g., (22, −5, −23), (20, −17, −25)… (28, −7, −11). These 125 points were mapped onto various brain regions using the Brainnetome Atlas (Fan et al., 2016), which can facilitate investigation of structure-function relationships and comparative neuroanatomical studies. The Brainnetome Atlas currently contains 246 regions of the bilateral hemispheres. Moreover, the atlas connectivity-based parcellation-yielded regions are functionally defined according to behavioral domain and paradigm class meta-data labels of the BrainMap database^3^ using forward and reverse inferences. The brain regions corresponding to the 125 points in the standard MNI space were located with the help of the Brainnetome Atlas. In this way, the brain regions with particularly significant contributions to the classification were identified according to the number of intersection points located in those regions. Here, the number of the intersection points determined by the most discriminable slices separating two classes in a binary classification task among the sagittal, coronal, and transverse slice sets, transformed into the standard MNI space, acted as an indicator to assess the contributions of a brain region in which the points were located to classifying AD. Given that the brain regions in a discriminable slice contribute to the classification of AD, we cannot deny the fact that a brain region at which an intersection point formed by three discriminable sagittal, coronal, and transverse slices is located contributes most to the classification of AD among all the brain regions that existed in the sagittal, coronal or transverse discriminable slice since the brain region exists in the three slices at the same time.

**FIGURE 8:**
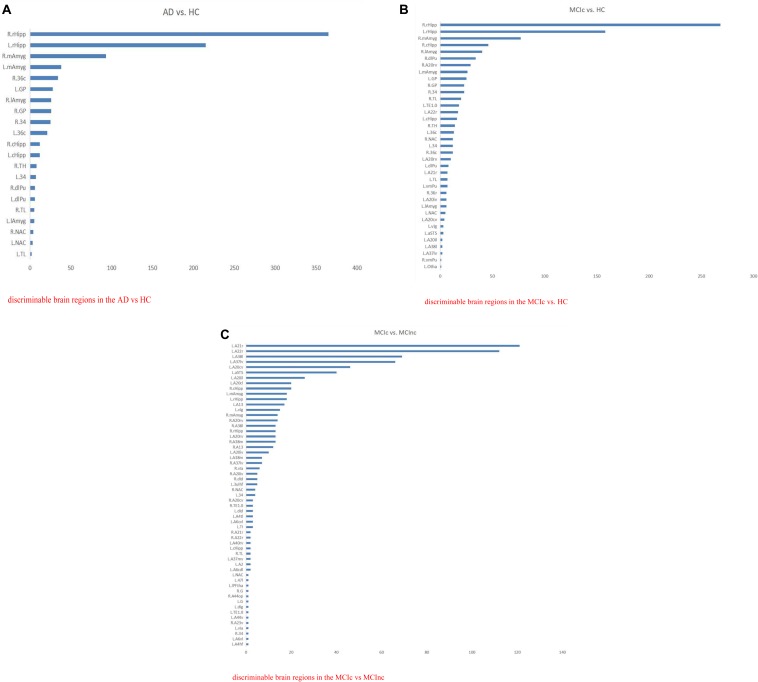
The list of brain regions with the classification capacity in each classification task. **(A)** Discriminable brain regions in the AD vs. HC. **(B)** Discriminable brain regions in the MCIc vs. HC. **(C)** Discriminable brain regions in the MCIc vs. MCInc.

In this way, for all the 10 × 5-fold cross-validation processes, the number of all the intersection points located in the same brain region is summed to measure the ability of the brain region to classify AD. The brain regions identified with the most intersection points might be the most discriminable for a binary classification task. Thus, the details of the identified brain regions with the classification capacity are shown in Figure 8 and Tables 8a–c. It is notable that the sum of the last column (i.e., the number of points located in a brain region) in each of the three tables was less than 1250 since some intersection points were located in the unlabeled brain regions. In Figure 8, values on the vertical and the horizontal axes represent the brain region labels and the number of intersection points located in each brain region, respectively. The prefix capital letters R and L of a brain region label (e.g., R.rHipp) refer to the right and left cerebral hemispheres, respectively.

**TABLE 8a:** Details of the discriminable brain regions in the AD vs. HC task.

Label of a brain region	Name of a brain region	# of points located in a brain region
R.rHipp	Rostral hippocampus	365
L.rHipp	Rostral hippocampus	215
R.mAmyg	Medial amygdala	93
L.mAmyg	Medial amygdala	38
R.36c	Caudal area 35/36	34
L.GP	Globus pallidus	28
R.GP	Globus pallidus	26
R.lAmyg	Lateral amygdala	26
R.34	Area 28/34 (EC, entorhinal cortex)	25
L.36c	Caudal area 35/36	21
L.cHipp	Caudal hippocampus	12
R.cHipp	Caudal hippocampus	12
R.TH	Area TH (medial PPHC)	8
L.34	Area 28/34 (EC, entorhinal cortex)	7
L.dlPu	Dorsolateral putamen	6
R.dlPu	Dorsolateral putamen	6
L.lAmyg	Lateral amygdala	5
R.TL	Area TL (lateral PPHC, posterior parahippocampal gyrus)	5
R.NAC	Nucleus accumbens	4
L.NAC	Nucleus accumbens	3
L.TL	Area TL (lateral PPHC, posterior parahippocampal gyrus)	2

**TABLE 8b:** Details of the discriminable brain regions in the MCIc vs. HC task.

Label of a brain region	Name of a brain region	# of points located in a brain region
R.rHipp	Rostral hippocampus	268
L.rHipp	Rostral hippocampus	158
R.mAmyg	Medial amygdala	77
R.cHipp	Caudal hippocampus	46
R.lAmyg	Lateral amygdala	40
R.dlPu	Dorsolateral putamen	34
R.A20rv	Rostroventral area 20	29
L.mAmyg	Medial amygdala	26
L.GP	Globus pallidus	25
R.34	Area 28/34 (EC, entorhinal cortex)	23
R.GP	Globus pallidus	23
R.TL	Area TL (lateral PPHC, posterior parahippocampal gyrus)	20
L.TE1.0	TE1.0 and TE1.2	18
L.A22r	Rostral area 22	17
L.cHipp	Caudal hippocampus	16
R.TH	Area TH (medial PPHC)	14
L.36c	Caudal area 35/36	13
R.36c	Caudal area 35/36	12
L.34	Area 28/34 (EC, entorhinal cortex)	12
R.NAC	Nucleus accumbens	12
L.A20rv	Rostroventral area 20	10
L.dlPu	Dorsolateral putamen	8
L.vmPu	Ventromedial putamen	7
L.TL	Area TL (lateral PPHC, posterior parahippocampal gyrus)	7
L.A21r	Rostral area 21	7
L.lAmyg	Lateral amygdala	6
L.A20iv	Intermediate ventral area 20	6
R.36r	Rostral area 35/36	6
L.NAC	Nucleus accumbens	5
L.A20cv	Caudoventral of area 20	4
L.aSTS	Anterior superior temporal sulcus	3
L.vIg	Ventral dysgranular and granular insula	3
L.A37lv	Lateroventral area 37	2
L.A38l	Lateral area 38	2
L.A20il	Intermediate lateral area 20	2
L.Otha	Occipital thalamus	1
R.vmPu	Ventromedial putamen	1

**TABLE 8c:** Discriminable brain regions in the MCIc vs. MCInc classification task.

Label of a brain region	Name of a brain region	# of points located in a brain region
L.A21r	Rostral area 21	121
L.A22r	Rostral area 22	112
L.A38l	Lateral area 38	69
L.A37lv	Lateroventral area 37	66
L.A20cv	Caudoventral of area 20	46
L.aSTS	Anterior superior temporal sulcus	40
L.A20il	Intermediate lateral area 20	26
R.cHipp	Caudal hippocampus	20
L.A20cl	Caudolateral of area 20	20
L.rHipp	Rostral hippocampus	18
L.mAmyg	Medial amygdala	18
L.A13	Area 13	17
L.vIg	Ventral dysgranular and granular insula	15
R.A20rv	Rostroventral area 20	14
R.mAmyg	Medial amygdala	14
R.A38m	Medial area 38	13
L.A20rv	Rostroventral area 20	13
R.rHipp	Rostral hippocampus	13
R.A38l	Lateral area 38	13
R.A13	Area 13	12
L.A20iv	Intermediate ventral area 20	10
R.A37lv	Lateroventral area 37	7
L.A38m	Medial area 38	7
R.vIa	Ventral agranular insula	6
L.3ulhf	Area 1/2/3 (upper limb, head and face region)	5
R.dId	Dorsal dysgranular insula	5
R.A20iv	Intermediate ventral area 20	5
L.34	Area 28/34 (EC, entorhinal cortex)	4
R.NAC	Nucleus accumbens	4
L.TI	Area TI (temporal agranular insular cortex)	3
L.A6cvl	Caudal ventrolateral area 6	3
L.A4tl	Area 4 (tongue and larynx region)	3
L.dId	Dorsal dysgranular insula	3
R.TE1.0	TE1.0 and TE1.2	3
R.A20cv	Caudoventral of area 20	3
L.A6cdl	Caudal dorsolateral area 6	2
L.A2	Area 1/2/3 (tongue and larynx region)	2
L.A37mv	Medioventral area 37	2
R.TL	Area TL (lateral PPHC, posterior parahippocampal gyrus)	2
L.cHipp	Caudal hippocampus	2
L.A40rv	Rostroventral area 40 (pfop)	2
R.A22r	Rostral area 22	2
R.A21r	Rostral area 21	2
L.A4hf	Area 4 (head and face region)	1
L.A6vl	Ventrolateral area 6	1
R.34	Area 28/34 (EC, entorhinal cortex)	1
L.vIa	Ventral agranular insula	1
R.A23v	Ventral area 23	1
L.A44v	Ventral area 44	1
L.TE1.0	TE1.0 and TE1.2	1
L.dIg	Dorsal granular insula	1
L.G	Hypergranular insula	1
R.A44op	Opercular area 44	1
R.G	Hypergranular insula	1
L.lPFtha	Lateral pre-frontal thalamus	1
L.47l	Lateral area 12/47	1
L.NAC	Nucleus accumbens	1

**FIGURE 9:**
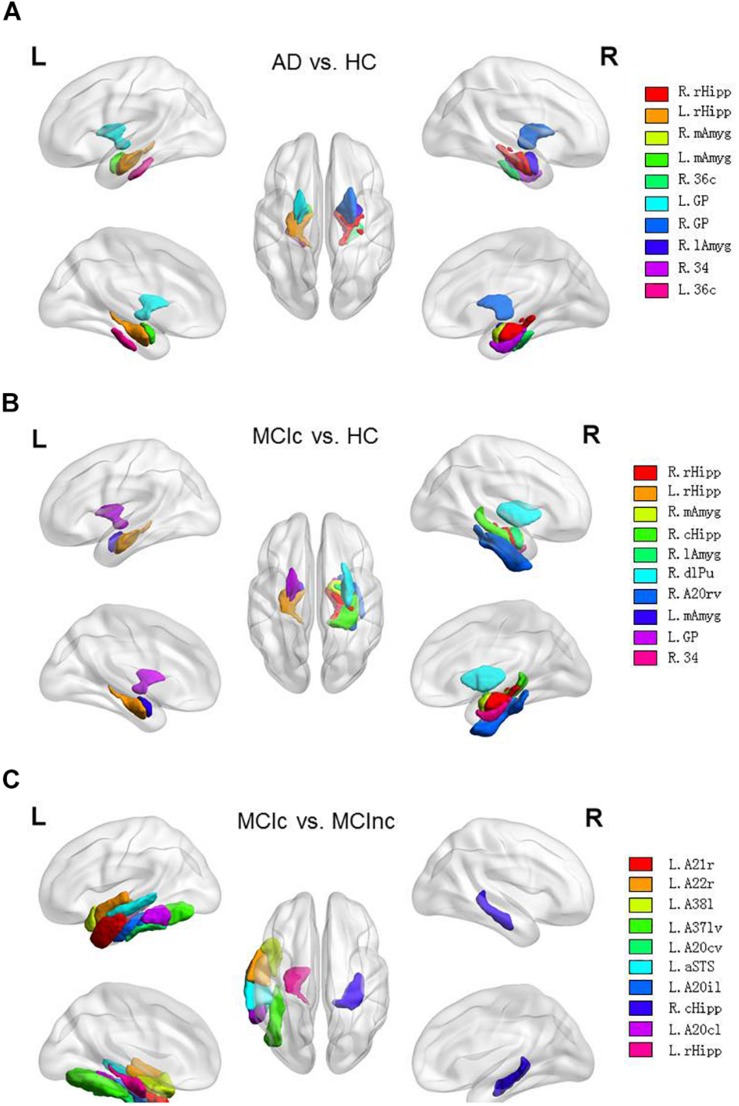
Top 10 most discriminable brain regions in each binary classification task: **(A)** AD vs. HC; **(B)** MCIc vs. HC; **(C)** MCIc vs. MCInc.

From the above figures and tables, the most discriminable brain regions in the AD vs. HC classification task were the rostral hippocampus (Greene et al., 2012), medial amygdala (Nelson et al., 2018), globus pallidus (Baloyannis, 2006), lateral amygdala (Kile et al., 2009), area 28/34 (EC, entorhinal cortex), and caudal area 35/36, i.e., parahippocampal gyrus (van Hoesen et al., 2000), while those in the MCIc vs. HC classification task were rostral hippocampus (Ighodaro et al., 2015), medial amygdala (Cavedo et al., 2014), caudal hippocampus (Chen et al., 2015), lateral amygdala (Kile et al., 2009), dorsolateral putamen (Reeves et al., 2010), rostroventral area 20, i.e., Fusiform gyrus (Bokde et al., 2006), globus pallidus (Hernández et al., 2020), area 28/34 (EC, entorhinal cortex) (Du et al., 2001; Burggren et al., 2011; Tward et al., 2017), and area TL (lateral PPHC, posterior parahippocampal gyrus) (Devanand et al., 2007). Finally, the most discriminable brain regions in the MCIc vs. MCInc classification task were rostral area 21 and anterior superior temporal sulcus, i.e., middle temporal gyrus (Karas et al., 2008); rostral area 22 and lateral area 38, i.e., superior temporal gyrus (Karas et al., 2008); lateroventral area 37, i.e., fusiform gyrus (Guillozet et al., 2003); and caudoventral of area 20 and intermediate lateral area 20 and caudolateral of area 20, i.e., inferior temporal gyrus (Scheff et al., 2011) and caudal hippocampus (Thomann et al., 2012). The top 10 most discriminable brain regions are mapped onto brain images in Figure 9.

In the paper (Yang et al., 2019), the results showed that the patients with aMCI (elderly patients with amnestic MCI) merely had slight atrophy in the inferior parietal lobe of the left hemisphere but a significant difference was NOT found in comparison with the NC (normal controls). The results are consistent with the highly lateralized MCIc vs. MCInc-related features acquired in this study, to some degree. Plus, the most discriminable brain regions identified in the MCIc vs. MCInc classification task in our study were in agreement with the conclusion of the paper (Yang et al., 2019) that the atrophy of cortical thickness and surface area in aMCI began in the temporal lobe but the range of atrophy gradually expanded with the progression of disease, to a great extent. Furthermore, in the paper (Karas et al., 2008), the obtained results were that MCI converters (patients with MCI who will progress to AD) had more left lateral temporal lobe atrophy (superior and middle temporal gyrus) and left parietal atrophy (angular gyrus and inferior parietal lobule) than MCI non-converters, i.e., stable patients with MCI, and the drawn conclusion was that by studying two MCI converter vs. non-converter populations, atrophy beyond the medial temporal lobe was found to be characteristic of converters and atrophy of structures such as the left parietal cortex and left lateral temporal lobe might independently predict conversion. The results and conclusion were consistent with most of our results to some extent.

**FIGURE 10:**
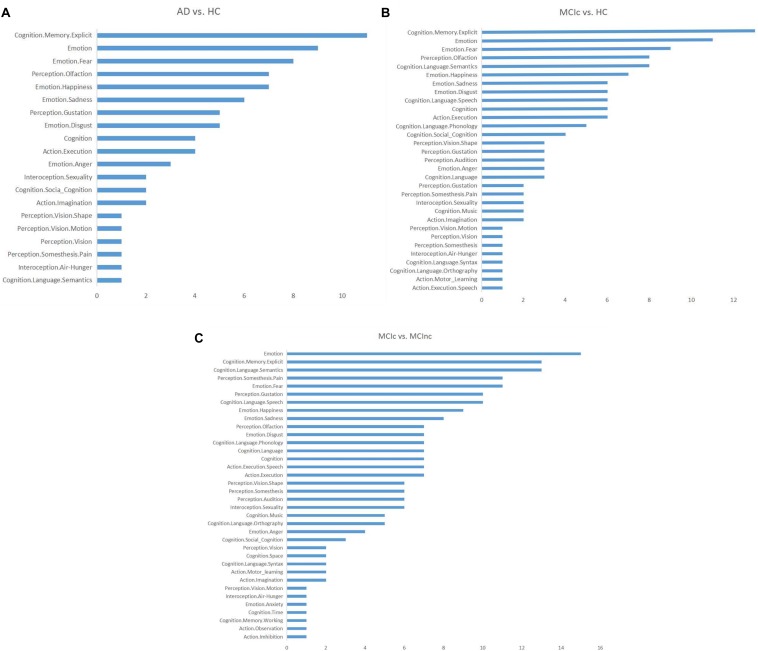
Distributions of the identified brain regions on the relevant behavioral domains in each binary classification task: **(A)** AD vs. HC; **(B)** MCIc vs. HC; **(C)** MCIc vs. MCInc.

After location mapping, the corresponding behavioral domains to every identified brain region were obtained from the Brainnetome Atlas official website,^4^ and the functions of these identified brain regions were analyzed. Then, the number of identified brain regions corresponding to each AD-related behavioral domain was calculated for each task (Figure 10) to reveal the distribution of structures showing the largest differences between classes and thus most informative for classification (e.g., emotion-related structures for AD vs. HC). In the figure, the vertical and horizontal axes show the relevant behavioral domains and the number of identified brain regions associated with these relevant behavioral domains, respectively.

From Figure 10, it can be seen that the functions related to these identified brain regions with the discriminability were mainly involved with the behavioral domains of emotion, memory, language, perception, internal feelings, and activity. The most common symptoms of AD, especially in the early stage, include memory loss that disrupts daily life, challenges in planning or problem solving, difficulty completing familiar tasks at home, at work, or at leisure, confusion with time or place, trouble understanding visual images and spatial relationships, new problems with words in speaking or writing, misplacing things and losing the ability to retrace steps, decreased or poor judgment, and changes in mood and personality (Mantzavinos and Alexiou, 2017). Thus, the behavioral domains relevant to the identified brain regions were generally consistent with the common symptoms of AD.

## Discussion

In this study, we developed a novel deep learning approach that combined CNN and EL and applied it to the most commonly acquired anatomical MRI of the brain, i.e., T1WI. We aimed to achieve two objectives: i.e., classification of AD or MCIc vs. HC, and MCIc vs. MCInc and identification of the complex change patterns associated with AD.

In comparison with a previous PCA plus SVM method (Christian et al., 2015), the current method does not require manual selection of ROIs, but automatically extracts the discriminable features from the MR images using a CNN-based adaptive representation learning method in a data-driven way. The proposed method employs a two-stage EL scheme to improve generalization and robustness. The model achieved average classification accuracies (± standard deviation) of 0.84 ± 0.05 for AD vs. HC, 0.79 ± 0.04 for MCIc vs. HC, and 0.62 ± 0.06 for MCIc vs. MCInc. Compared to the PCA plus SVM method, the proposed method showed statistically substantially improved accuracy and robustness for distinguishing among the AD, MCIc, and HC groups, while model accuracy was NOT statistically lower than that achieved by the PCA plus SVM method for distinguishing MCIc from MCInc. At the same time, compared to the 3D-SENet model, the CNN-EL method achieved statistically higher accuracy and robustness for all the three binary classification tasks.

For a 1 × 5-fold cross-validation processes, we also identified the 15 slices and resultant 125 (i.e., 5 × 5 × 5) intersection points in the standard MNI space based on the five best base classifiers trained respectively with sagittal, coronal, or transverse slice data. These points were then mapped onto the Brainnetome Atlas to identify the corresponding brain regions with the discriminability in the three binary classification tasks. For all the 10 × 5-fold cross-validation processes, the number of all the intersection points located in the same brain region was summed to evaluate the capability of the brain region to help diagnose AD. The identified brain regions included hippocampus, amygdala, and temporal lobe, which are known to be affected by AD and involved in neurological processes impaired in AD (Schroeter et al., 2009). Also, we acquired the corresponding behavioral domains based on all identified brain regions, which were generally consistent with the common symptoms of AD.

In two-dimensional convolutional neural network (2D-CNN)-based models for early detection of AD, only sagittal, coronal, or transverse slices of 3D MR images are usually used as the training dataset. A specific slice, such as a transverse slice through the hippocampus, was often selected based on experience or prior domain knowledge (Wang et al., 2018). Using only the data from a single 2D slice of a 3D MR image removes potentially valuable information. In comparison, the novel CNN-EL approach that we proposed here has the following significant features:

(1) Six data augmentation (DA) methods are used to deal with the imbalanced data problem by disproportionately increasing the number of image slices in classes with fewer samples. As a result, each class can have approximately an equal increased number of training instances in the augmented dataset.
(2) The proposed ensemble model combines features identified from the sagittal, coronal, and transverse slices of a 3D MRI dataset together, to improve classification accuracy and model adaptability. Each of the base 2D CNN classifier was trained with the data from a single slice orientation. Then, the top “N” trained base classifiers were selected according to the generalization performance on the verification dataset to build the final ensemble. In this way, the method effectively improved classification accuracy and robustness. The slices used as training data to construct base classifiers were not necessarily specified based on prior domain knowledge; rather, each available and valid slice (sagittal, coronal, or transverse) in the dataset was used to train the corresponding base classifier.
(3) Compared to the length of time spent on building a model with data from only a single slice orientation, it may take more time to build the proposed model since many more base classifiers need to be trained. To effectively solve this problem, the parallel processing method was adopted to train the base classifiers used to build the ensemble model. This greatly improved the training efficiency and made the proposed model scalable.
(4) According to the classification performances of all trained base classifiers on the verification dataset, the three sets of top “N” base classifiers trained using data from sagittal, coronal, and transverse slices, respectively, were determined. Since a base classifier was trained with the data from only a specific slice orientation, the most important sagittal, coronal, or transverse slice for a binary classification task (e.g., AD vs. HC) could be located according to the three sets of top “N” base classifiers in a data-driven way. Furthermore, the brain regions corresponding to the intersection points determined by the top “N” sagittal, coronal, and transverse slices could be located with the help of the Brainnetome Atlas. The brain regions identified with the most intersection points might be the most discriminable for a binary classification task, given that the number of the intersection points could be an indicator to measure the ability of a brain region in which the points were located to classify AD.
(5) The performance of the proposed classifier ensemble was compared to that of other machine learning models using the same dataset. The experimental results showed that the proposed model achieved better classification accuracy and robustness.

The relatively low classification accuracy for MCIc vs. MCInc warrants further investigation and the classification performance needs to be improved with the optimization methods and/or other deep learning models to identify the brain regions with stronger discriminability.

For an individual subject to be diagnosed, the votes of base classifiers in the trained classifier ensemble based on the three-axis slices and the number of resulting intersection points located in each brain region might be employed to disclose the extent to which AD impaired each brain region and each behavioral domain, which could help understand and evaluate the subject’s disease status, symptom burden and, more importantly, progress. Plus, with the advancements of brain atlases and advanced ultra-high-field scanners, chances are that the positions and the number of the intersection points determined by the proposed CNN-EL methods might provide more details on and insights into the progress of AD pathology.

Furthermore, the advocated method may be useful for identifying additional candidate neuroimaging biomarkers for AD as well as for other brain diseases such as Parkinson’s disease, autism, schizophrenia and severe depression, especially for identifying candidate neuroimaging biomarkers for other little-known brain disorders, in a data-driven way.

The above-mentioned discussions, the clinical implication of the finding applying other samples, and the generalizability of the advocated CNN-EL approach need to be examined in the future research.

## Data Availability Statement

The datasets analyzed for this study can be found in the Alzheimer’s Disease Neuroimaging Initiative (ADNI) database (adni.loni.usc.edu).

## Author Contributions

DP and AZ designed and coordinated the study. LJ, DP, YH, and AZ carried out experiment and data process. XS reviewed the study design and data processing, and edited results interpretation and presentation. All authors drafted and revised the manuscript, and approved the final version of the submitted manuscript.

## Conflict of Interest

The authors declare that the research was conducted in the absence of any commercial or financial relationships that could be construed as a potential conflict of interest.

## Footnotes

1 adni.loni.usc.edu

2 http://dbm.neuro.uni-jena.de/cat/

3 http://www.brainmap.org/taxonomy

4 www.Brainnetome.org
